# Is Combined PhacoAhmed Less Effective than Ahmed Surgery Alone? A 5-Year Retrospective Study of Long-Term Effects

**DOI:** 10.3390/vision9030068

**Published:** 2025-08-04

**Authors:** Maria Vivas, José Charréu, Bruno Pombo, Tomás Costa, Ana Sofia Lopes, Fernando Trancoso Vaz, Maria João Santos, Isabel Prieto

**Affiliations:** 1 Ophtalmology Department, Hospital Professor Doutor Fernando Fonseca, 2700 Lisbon, Portugal; 2 Glaucoma Department, Hospital Professor Doutor Fernando Fonseca, 2700 Lisbon, Portugal

**Keywords:** PhacoAhmed, Ahmed, glaucoma, drainage device, FP7

## Abstract

Combined trabeculectomy–phacoemulsification is known to provoke more inflammation and yield a poorer long-term efficacy than trabeculectomy alone. This study evaluates whether a similar trend exists for Ahmed glaucoma valve implantation when performed with or without concurrent phacoemulsification. We retrospectively analyzed 51 eyes from patients who underwent either Ahmed-Alone (n = 25) or PhacoAhmed (n = 26) surgery over a 5-year period. The primary outcomes included intraocular pressure (IOP), the use of IOP-lowering medications, and the need for further surgical intervention. Absolute success was defined as IOP reduction > 20% and IOP < 21 mmHg without medication; relative success allowed for continued pharmacologic therapy. Both groups showed a significant IOP reduction, with similar final mean IOP values (Ahmed-Alone: 14.02 ± 4.76 mmHg; PhacoAhmed: 13.89 ± 4.17 mmHg; *p* = 0.99) and comparable reductions in medication use (*p* = 0.52). Reinterventions occurred less frequently and later in the PhacoAhmed group (12% vs. 27.3%; median time: 27.1 vs. 12 months). Absolute success was not achieved in any PhacoAhmed case but occurred in 9.3% of Ahmed-Alone cases; relative success rates were similar (83.3% vs. 81.4%; *p* = 0.291). These findings suggest that combining phacoemulsification with Ahmed valve implantation does not significantly alter efficacy or safety profiles. Additional prospective studies are warranted to assess long-term outcomes.

## 1. Introduction

Glaucoma is the leading cause of irreversible blindness worldwide, characterized by optic nerve damage and visual field loss. Its management often involves pharmacological treatment, laser therapy, and surgical interventions to control intraocular pressure (IOP) and prevent disease progression.

The Ahmed Glaucoma Valve (AGV) is widely used for its effectiveness in lowering IOP, particularly in cases with a history of failed filtering surgery or in conditions such as uveitic or neovascular glaucomas, which are known to result in higher failure rates of trabeculectomy [1].

Historically, drainage devices were initially placed in the anterior chamber, especially when it was sufficiently deep, to manage IOP. However, this approach resulted in corneal complications in 2–27% of cases [2]. To mitigate these issues, the AGV tube can be placed into the ciliary sulcus, particularly during cataract surgery or in pseudophakic patients [3].

Additionally, cataract development—common in glaucoma patients—presents challenges, as both conditions impact vision and quality of life. However, the optimal approach for combining cataract surgery with glaucoma procedures remains debated.

It is also documented that phacoemulsification in patients with a prior history of recent trabeculectomy (often less than 6 months) results in less-favorable surgical outcomes compared to trabeculectomy alone [4]. Additionally, trabeculectomy performed in combination with phacoemulsification yields inferior outcomes relative to trabeculectomy as a standalone procedure [5,6]. This raises the question as to whether similar trends apply to the Ahmed Glaucoma Valve.

Considering this, our study evaluates and compares the outcomes of the Ahmed Glaucoma Valve as a standalone procedure versus its combination with cataract surgery, contributing to better-informed treatment decisions for coexisting glaucoma and cataracts.

## 2. Materials and Methods

We present a retrospective study including all patients who underwent the Ahmed^®^ Glaucoma Valve Model FP7 implantation, either as a standalone procedure or combined with phacoemulsification, over the past 5 years, reviewing all medical records of patients that had at least 5 years of follow-up. The statistical analysis was performed using IBM SPSS Statistics software^®^ (Version 31.0.0.0).

For all patients, the analysis included baseline (BL) intraocular pressure (IOP), along with IOP measurements measured with a Goldmann Applanation Tonometer at 1 week (1W), 1 month (1M), 3 months (3M), 6 months (6M), 1 year (1Y), and then annually (Years 2, 3, 4, and 5), as well as the final follow-up observation (FF).

We also assessed the number of glaucoma medications at baseline, as well as at 1 month, 3 months, 6 months, 1 year, and at the final follow-up.

Additionally, we evaluated the incidence of and the need for further surgical interventions, such as needling, trephination, implantation of additional drainage devices, or cyclophotocoagulation procedures (using a CycloG6^®^ Laser with MicroPulse P3^®^ Delivery Device). The time to these events was recorded to better understand postoperative outcomes and complications.

Regarding outcomes, such as surgical efficacy, absolute success was defined according to the World Glaucoma Association as >20% IOP reduction and IOP < 21 mmHg without medication; relative success was defined as >20% reduction and IOP < 21 mmHg with medication; and failure was defined as <20% IOP reduction and/or IOP > 21 mmhg.

Independent samples were analyzed corresponding to two distinct groups of patients—one group underwent standalone Ahmed valve implantation (Ahmed-Alone) and the other underwent combined implantation with phacoemulsification (PhacoAhmed). Each group was independently assessed at various postoperative time points. To compare continuous variables (e.g., IOP and the number of hypotensive medications) between the two groups, the independent samples *t*-test was used (or the Mann–Whitney U test if the data did not follow a normal distribution). For categorical variables, such as rates of absolute success, relative success, and failure, the chi-square test was employed. Data were presented as means ± standard deviations (or medians and interquartile ranges for non-parametric data), and 95% confidence intervals were calculated. A *p*-value < 0.05 was considered statistically significant. We obtained informed consent from all patients included in this study.

## 3. Results

A total of 51 eyes from 49 patients were analyzed. Of these, 26 eyes underwent Ahmed^®^ FP7 Glaucoma Valve implantation combined with phacoemulsification (the PhacoAhmed group) and 25 eyes underwent standalone AGV implantation (the Ahmed-Alone group). The mean patient age was 69.02 ± 13.39 years, and the average follow-up period was 5.78 years. At baseline, the mean deviation (MD) was −11.3 ± 5.4 dB in the Ahmed-Alone group and −10.7 ± 4.9 dB in the PhacoAhmed group (*p* = 0.63), indicating a comparable degree of functional visual field loss between cohorts.

### 3.1. Glaucoma Types

The distribution of glaucoma types was analyzed between the PhacoAhmed group (n = 26) and the Ahmed-Alone group (n = 25) (Table 1). In the PhacoAhmed group, primary open-angle glaucoma (POAG) accounted for 61.5% of cases (n = 16), neovascular glaucoma (NVG) for 23.1% (n = 6), pseudoexfoliative glaucoma (PXG) for 7.7% (n = 2), and uveitic glaucoma for 7.7% (n = 2). No cases of traumatic glaucoma were observed in this group. In the Ahmed-Alone group, POAG represented 76.0% of cases (n = 19), NVG 12.0% (n = 3), PXG 4.0% (n = 1), uveitic glaucoma 4.0% (n = 1), and traumatic glaucoma 4% (n = 1). The differences in glaucoma type distribution between the two groups were not statistically significant (*p* = 0.619).

### 3.2. Intraocular Pressure (IOP)

**Figure 1:**
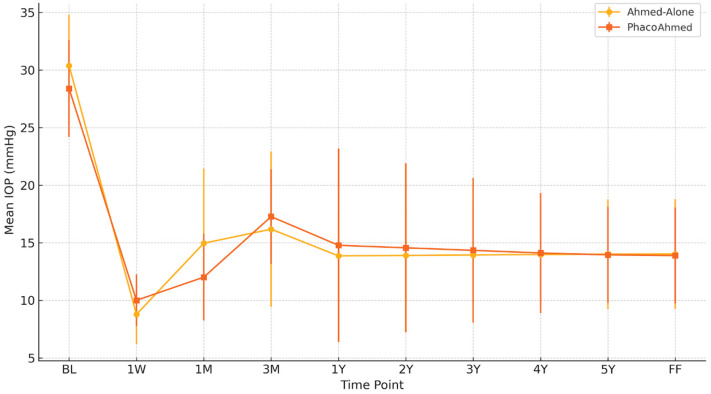
Comparison of intraocular pressure (IOP) between the Ahmed-Alone and PhacoAhmed groups across time points.

○ Baseline IOP was similar between the groups, with the PhacoAhmed group recording a mean IOP of 28.39 ± 4.2 mmHg and the Ahmed-Alone group recording a value of 30.39 ± 4.4 mmHg (Table 2 and Figure 1). At 1W post operation, the mean IOP value was 8.78 ± 2.60 mmHg for the Ahmed-Alone (AA) group and 10 ± 2.25 mmHg for the PhacoAhmed group. After 1M, it was 14.96 ± 6.49 mmHg for the Ahmed-Alone group and 12.01 ± 3.76 mmHg for the PhacoAhmed group. After that, it was 16.18 ± 6.75 mmHg and 17.27 ± 4.12 mmHg, respectively, for the 3M evaluation, and 13.86 ± 3.63 mmHg and 14.78 ± 8.40 mmHg for the 1Y postoperative observation. The annual IOP estimates demonstrated temporal stability with no significant variation observed between Years 2 and 5 for both the Ahmed-Alone and PhacoAhmed groups, respectively, at 13.90 ± 3.91 mmHg vs. 14.56 ± 7.34 mmHg (Year 2); 13.94 ± 4.20 vs. 14.34 ± 6.28 mmHg (Year 3); 13.98 ± 4.48 vs. 14.11 ± 5.23 mmHg (Year 4); and 14.01 ± 4.76 mmHg vs. 13.95 ± 4.17 mmHg (Year 5).
○ At FF time, the mean IOP value obtained for the Ahmed-Alone group was 14.02 ± 4.76 mmHg vs. 13.89 ± 4.17 mmHg in the PhacoAhmed group, with no statistical significance (*p*-value = 0.99).

**Table 2:** Comparison of intraocular pressure (IOP) between the Ahmed-Alone and PhacoAhmed groups across time points.

Time Point	Ahmed-Alone (Mean IOP ± SD)	PhacoAhmed (Mean IOP ± SD)	*p*-Value
BL	30.39 ± 4.4 mmHg	28.39 ± 4.2 mmHg	0.9
1W	8.78 ± 2.60 mmHg	10 ± 2.25 mmHg	0.19
1M	14.96 ± 6.49 mmHg	12.01 ± 3.76 mmHg	0.27
3M	16.18 ± 6.75 mmHg	17.27 ± 4.12 mmHg	0.83
1Y	13.86 ± 3.63 mmHg	14.78 ± 8.40 mmHg	0.88
2Y	13.90 ± 3.91 mmHg	14.56 ± 7.34 mmHg	0.91
3Y	13.94 ± 4.20 mmHg	14.34 ± 6.28 mmHg	0.94
4Y	13.98 ± 4.48 mmHg	14.11 ± 5.23 mmHg	0.96
5Y	14.01 ± 4.76 mmHg	13.95 ± 4.17 mmHg	0.99
FF	14.02 ± 4.76 mmHg	13.89 ± 4.17 mmHg	0.99

### 3.3. Number of IOP-Lowering Drops

The baseline number of IOP-lowering drops in the Ahmed-Alone group had a mean value of 3.74 ± 0.57, while the PhacoAhmed group recorded a value of 3.58 ± 0.67 (comparable between groups; Table 3). One month post-intervention, the mean number of medications was 2.37 ± 0.94 for the Ahmed-Alone group and 2.75 ± 1.06 for the PhacoAhmed group. At 3 months, the values were 2.21 ± 1.09 and 2.58 ± 1.00, respectively. By 6 months, the mean number of drops was 2.21 ± 1.17 for the Ahmed-Alone group and 2.75 ± 1.06 for the PhacoAhmed group. At 1 year, values were 2.22 ± 1.15 for the Ahmed-Alone group and 2.76 ± 1.01 for the PhacoAhmed group. In subsequent annual follow-ups, the Ahmed-Alone group maintained relatively stable values, as follows: 2.24 ± 1.17 at Year 2, 2.27 ± 1.16 at Year 3, 2.30 ± 1.16 at Year 4, and 2.31 ± 1.10 at Year 5. The PhacoAhmed group showed similarly stable values over time, with 2.72 ± 1.06 at Year 2, 2.71 ± 1.05 at Year 3, 2.72 ± 1.03 at Year 4, and 2.73 ± 1.06 at Year 5.

Need for subsequent Needling and/or Trephination

Early or late bleb fibrosis can reduce filtration channel efficacy, leading to suboptimal intraocular pressure (IOP) control and surgical failure.

Needling procedures were required in 19.2% of cases (n = 5) in the PhacoAhmed group and 16% (n = 4) in the Ahmed-Alone group (*p* = 1.0). The median time to needling was longer in the PhacoAhmed group, at 8.5 months compared to 4.6 months in the Ahmed-Alone group (*p* = 0.007).

Trephination was required in 20% of cases (n = 5) in the Ahmed-Alone group and 11.5% of cases (n = 3) in the PhacoAhmed group (*p* = 0.465). The median time to trephination was longer in the PhacoAhmed group, at 17 months compared to 7.8 months in the Ahmed-Alone group (*p* = 0.014).

Need for additional procedures

Further procedures, such as cyclophotocoagulation or the implantation of additional drainage devices, became necessary due to the inability to achieve adequate IOP control. Cyclophotocoagulation was mainly reserved for eyes with significantly reduced visual acuity (<1/10—Snellen Scale), while the placement of a new valve was prioritized for eyes with a greater visual acuity.

In total, 12% of the PhacoAhmed group compared to 27.3% of the Ahmed-Alone group needed a new procedure, but the median time to reintervention was longer in the PhacoAhmed group (27.1 months) than in the Ahmed-Alone group (12 months) (*p*-value = 0.008). Among those who underwent a new procedure, 7% in the PhacoAhmed group required a new drainage device compared to 20% in the Ahmed-Alone group. However, this difference was not statistically significant (*p*-value = 0.455).

Success Rates

Surgical success was categorized for each group. Absolute Success was defined as an IOP reduction > 20% with IOP < 21 mmHg without medications; relative success was defined as an IOP reduction > 20% with IOP < 21 mmHg with medications; and failure was defined as an IOP reduction < 20% or sustained IOP > 21 mmHg despite maximum medical therapy.

In the PhacoAhmed group (n = 26), 83.3% of eyes achieved relative success, while 16.7% were classified as failures. No cases of absolute success were observed in this group.

In the Ahmed-Alone group (n = 25), 81.4% of eyes achieved relative success, 9.3% achieved absolute success, and 9.3% were classified as failures. The differences in the success rates between the two groups were not statistically significant (*p* = 0.291).

**Figure 2:**
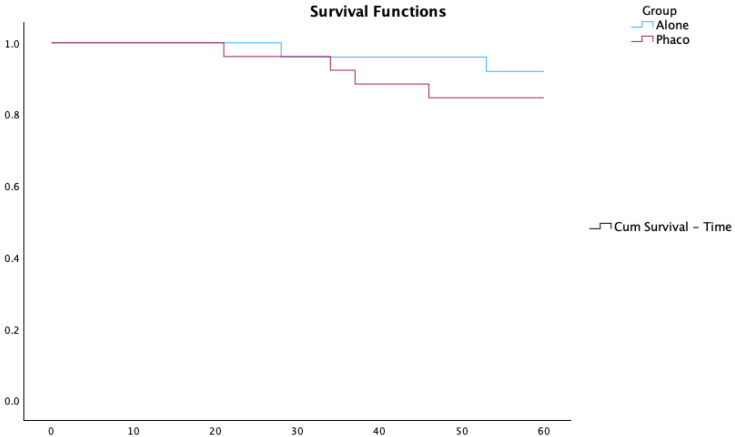
Kaplan–Meier survival curves comparing the Ahmed-Alone and PhacoAhmed groups.

Kaplan–Meier survival curves comparing the Ahmed-Alone and PhacoAhmed groups are presented in Figure 2.

In accordance with the World Glaucoma Association (WGA) guidelines, we further analyzed the outcomes using multiple upper IOP thresholds to better reflect clinical success. When considering a target IOP ≤ 18 mmHg, 88.4% of eyes in the Ahmed-Alone group and 83.3% in the PhacoAhmed group achieved relative surgical success (*p*-value = 0.837). For a more stringent threshold of IOP ≤ 15 mmHg, 62.8% of Ahmed-Alone eyes and 75.0% of PhacoAhmed eyes met the success criteria (*p*-value = 0.454). These results offer a more nuanced evaluation of efficacy, which is particularly relevant for patients with advanced glaucomatous damage who may require lower target pressures.

## 4. Discussion

This study provides a comprehensive analysis of the outcomes associated with Ahmed Glaucoma Valve (AGV) implantation, either as a standalone procedure or combined with phacoemulsification. Primary open-angle glaucoma (POAG) emerged as the most prevalent type of glaucoma in this study cohort, accounting for 68% of all cases. This finding reflects the trabeculectomy failure rate in this population and the broader epidemiological trends observed globally, where POAG represents the leading subtype of glaucoma, particularly in aging populations.

Our findings underscore important clinical insights that have implications for the management of glaucoma. Both groups (Ahmed-Alone and PhacoAhmed) demonstrated significant reductions in intraocular pressure (IOP) from baseline to final follow-up, with comparable mean IOP levels at all postoperative time points (Ahmed-Alone: 14.02 ± 4.76 mmHg; PhacoAhmed: 13.89 ± 4.17 mmHg; *p* = 0.99). This suggests that combining phacoemulsification with AGV implantation does not compromise the ability to achieve long-term IOP control, aligning with previous studies that report a comparable efficacy between standalone and combined procedures [7]. The number of IOP-lowering medications decreased in both groups postoperatively, but the Ahmed-Alone group consistently required fewer medications at all follow-up points, though the differences were not statistically significant. This suggests a slight advantage of standalone AGV implantation in reducing dependency on medications, which is consistent with findings from earlier studies [8].

The proportion of patients requiring needling was similar between the two groups (19.2% in the PhacoAhmed group vs. 16% in the Ahmed-Alone group; *p* = 1.0). This suggests that the type of procedure (standalone or combined with phacoemulsification) did not significantly influence the need for additional interventions to address bleb fibrosis. However, the median time to needling was significantly longer in the PhacoAhmed group (8.5 vs. 4.6 months; *p* = 0.007).

Similarly, the proportion of patients requiring trephination was higher in the Ahmed-Alone group (20% vs. 11.5%; *p* = 0.465), but the difference was not statistically significant. This lack of significance may be due to the small sample size, limiting the ability to detect meaningful differences. However, the median time to trephination was significantly longer in the PhacoAhmed group (17 vs. 7.8 months; *p* = 0.014).

Additionally, a higher percentage of the Ahmed-Alone group required additional procedures (27.3% vs. 12%; *p*-value = 0.13), and the median time to reintervention was shorter (12 months vs. 27.1 months in the PhacoAhmed group; *p*-value = 0.008).

While the combined procedure (PhacoAhmed) demonstrated a longer time to reintervention compared to the Ahmed-Alone group, this finding contradicts theoretical expectations and existing evidence suggesting increased inflammation with phacoemulsification. This discrepancy could potentially be explained by the different glaucoma profiles between the two populations; however, as observed in our study, the distribution of glaucoma types was similar across both groups, and the differences were not statistically significant. Therefore, the observed disparity in reintervention time, which does not align with typical findings from the literature, cannot be attributed to variations in glaucoma type between the groups.

One possible hypothesis is that since the AGV is designed with a valve mechanism to prevent early postoperative hypotony, this may result in a subsequent hypertensive phase characterized by an elevated IOP that can take occur over weeks or months and can prompt interventions such as needling, trephination, or other procedures to manage IOP levels.

Regarding success rates, in the PhacoAhmed group (n = 26), 83.3% of eyes achieved relative success (IOP reduction > 20% with IOP < 21 mmHg on medication), 16.7% were classified as failures (IOP reduction < 20% or sustained IOP > 21 mmHg despite maximum therapy), and no case reached absolute success (IOP reduction > 20% with IOP < 21 mmHg without medication). In the Ahmed-Alone group (n = 25), 81.4% achieved relative success, 9.3% achieved absolute success, and 9.3% were classified as failures. These differences were not statistically significant (*p* = 0.291). Our results align with the existing literature, as demonstrated by Kang et al. (2022) [9].

Overall, both the combined PhacoAhmed valve procedure and the standalone Ahmed surgery demonstrated similar rates of success, suggesting that adding phacoemulsification does not significantly alter postoperative IOP outcomes compared to Ahmed implantation alone.

While trabeculectomy alone has shown superior long-term outcomes compared to combined surgeries, the combined AGV–phacoemulsification approach appears to effectively balance IOP control and visual rehabilitation. Notably, the study contrasts with findings in trabeculectomy, where outcomes are often inferior when combined with cataract surgery [10].

This may be explained by the fact that the healing reaction in Ahmed tube implantation and trabeculectomy differs, primarily due to the presence of a foreign device in the former. The AGV implant, positioned in a created pocket between the sub-tenon and scleral spaces, leads to a fibrotic response around the implant, with the reaction being directed to the device material with capsule formation [10]. In contrast, trabeculectomy does not involve a foreign object and instead relies only on tissue healing, potentially resulting in excessive subconjunctival cicatrization that can block the filtering bleb and lead to failure [11]. As such, this may explain why the PhacoAhmed approach can be more successful in the long term compared to Phacotrabeculectomy.

One limitation of our study is the absence of systematic data regarding the exact location of tube placement (anterior chamber vs. ciliary sulcus) across all cases. While in the PhacoAhmed group all tubes were placed in the ciliary sulcus at the time of combined surgery, tube position in the Ahmed-Alone group was determined by lens status, i.e., in phakic eyes, tubes were typically placed in the anterior chamber, whereas in pseudophakic eyes, sulcus placement was preferred. Although tube location may influence long-term outcomes—particularly with regard to endothelial cell loss and safety profiles—this variable was not uniformly documented and therefore could not be analyzed. Future prospective studies should account for this factor when assessing comparative efficacy and complications. The retrospective nature of this study and the relatively small sample size may also limit its generalizability. Our study suggests that both standalone and combined AGV–phacoemulsification procedures effectively manage IOP in glaucoma patients. Moreover, although standalone AGV surgery may reduce medication dependence, the combined PhacoAhmed approach can be a reasonable option with observed similar success results and long-term efficacy. Prospective, multicenter, randomized controlled trials with larger sample sizes will be essential to confirm these findings and further investigate the role of new devices and treatments in optimizing long-term outcomes.

## 5. Conclusions

This 5-year retrospective study suggests that combining phacoemulsification with Ahmed Glaucoma Valve implantation (PhacoAhmed) does not significantly alter long-term intraocular pressure (IOP) control or reduce the need for medications compared to Ahmed surgery alone. While the PhacoAhmed group exhibited a longer time to reintervention, this difference was not statistically significant. These findings imply that both surgical approaches may offer comparable outcomes in managing glaucoma, highlighting the need for further prospective studies to confirm these results and refine surgical decision-making.

## Figures and Tables

**Table 1:** Distribution of glaucoma types between the PhacoAhmed and Ahmed-Alone groups.

Glaucoma Type	PhacoAhmed (n)	PhacoAhmed (%)	Ahmed-Alone (n)	Ahmed-Alone (%)
Primary Open-Angle Glaucoma (POAG)	16	61.5%	19	76.0
Neovascular Glaucoma (NVG)	6	23.1%	3	12.0
Pseudoexfoliative Glaucoma (PXG)	2	7.7%	1	4.0
Uveitic Glaucoma	2	7.7%	1	4.0
Traumatic Glaucoma	0	0%	1	4.0
*p*-value				0.619

**Table 3:** Comparison of the number of IOP-lowering drops between the Ahmed-Alone and PhacoAhmed groups across time points.

Time Point	Ahmed-Alone (Mean IOP-Lowering Drug ± SD)	PhacoAhmed (Mean IOP-Lowering Drug ± SD)	*p*-Value
BL	3.74 ± 0.57 drops	3.58 ± 0.67 drops	0.48
1M	2.37 ± 0.94 drops	2.75 ± 1.06 drops	0.53
3M	2.21 ± 1.09 drops	2.58 ± 1.00 drops	0.59
6M	2.21 ± 1.17 drops	2.75 ± 1.06 drops	0.34
1Y	2.22 ± 1.15 drops	2.76 ± 1.01 drops	0.084
2Y	2.24 ± 1.17 drops	2.72 ± 1.06 drops	0.38
3Y	2.27 ± 1.16 drops	2.71 ± 1.05 drops	0.43
4Y	2.30 ± 1.16 drops	2.72 ± 1.03 drops	0.48
5Y	2.31 ± 1.10 drops	2.73 ± 1.06 drops	0.176
FF	2.33 ± 1.16 drops	2.75 ± 1.06 drops	0.52

## Data Availability

The data supporting the findings of this study are available from the corresponding author upon reasonable request. No publicly archived datasets were analyzed or generated during the current study.
